# Fructose ingestion impairs expression of genes involved in skeletal muscle’s adaptive response to aerobic exercise

**DOI:** 10.1186/s12263-017-0588-9

**Published:** 2017-12-08

**Authors:** Natalia Gomes Gonçalves, Stephanie Heffer Cavaletti, Carlos Augusto Pasqualucci, Milton Arruda Martins, Chin Jia Lin

**Affiliations:** 1 Department of Pathology, School of Medicine, University of São Paulo, São Paulo, Brazil; 2 Department of Internal Medicine, School of Medicine, University of São Paulo, São Paulo, Brazil

**Keywords:** Fructose, Exercise training, Skeletal muscle, Protein turnover, PGC-1α, Rats

## Abstract

**Background:**

The inverse relationship between exercise capacity and its variation over time and both cardiovascular and all-cause mortality suggests the existence of an etiological nexus between cardiometabolic diseases and the molecular regulators of exercise capacity. Coordinated adaptive responses elicited by physical training enhance exercise performance and metabolic efficiency and possibly mediate the health benefits of physical exercise. In contrast, impaired expression of genes involved in mitochondrial biogenesis or protein turnover in skeletal muscle—key biological processes involved in adaptation to physical training—leads to insulin resistance and obesity. Ingestion of fructose has been shown to suppress the exercise-induced GLUT4 response in rat skeletal muscle. To evaluate in greater detail how fructose ingestion might blunt the benefits of physical training, we investigated the effects of fructose ingestion on exercise induction of genes that participate in regulation of mitochondrial biogenesis and protein turnover in rat’s skeletal muscle.

**Methods:**

Eight-week-old Wistar rats were randomly assigned to sedentary (C), exercise (treadmill running)-only (E), fructose-only (F), and fructose + exercise (FE) groups and treated accordingly for 8 weeks. Blood and quadriceps femoris were collected for biochemistry, serum insulin, and gene expression analysis. Expression of genes involved in regulation of mitochondrial biogenesis and autophagy, GLUT4, and ubiquitin E3 ligases MuRF-1, and MAFbx/Atrogin-1 were assayed with quantitative real-time polymerase chain reaction.

**Results:**

Aerobic training improved exercise capacity in both E and FE groups. A main effect of fructose ingestion on body weight and fasting serum triglyceride concentration was detected. Fructose ingestion impaired the expression of PGC-1α, FNDC5, NR4A3, GLUT4, Atg9, Lamp2, Ctsl, Murf-1, and MAFBx/Atrogin-1 in skeletal muscle of both sedentary and exercised animals while expression of Errα and Pparδ was impaired only in exercised rats.

**Conclusions:**

Our results show that fructose ingestion impairs the expression of genes involved in biological processes relevant to exercise-induced remodeling of skeletal muscle. This might provide novel insight on how a dietary factor contributes to the genesis of disorders of glucose metabolism.

## Background

The importance of physical activity as an essential component of a healthy lifestyle cannot be overlooked. Regular physical exercise enhances health span [1] while lack of physical activity or decreased physical fitness confers increased risk for premature death and increased risk for several chronic, non-communicable diseases [2]. Physical fitness or exercise capacity is a better predictor of mortality than traditional cardiovascular risk factors [3], and changes in exercise capacity over time are strong and inversely associated with all-cause mortality in men [4]. Interestingly, rats selectively bred for low exercise capacity exhibited defects characteristic of metabolic syndrome such as elevated blood pressure, impaired glucose tolerance, visceral adiposity, and elevated circulating levels of triglycerides [5]. These observations suggest that cardiometabolic diseases and the molecular determinants of exercise capacity are etiologically connected.

Physical exercise produces mechanical, metabolic, nutritional, and oxidative stresses in engaged skeletal muscles. These stimuli trigger a set of coordinated adaptive responses which result in modification of volume, protein content, mechanical properties, and metabolic capacities [6]. These responses restore homeostasis and improve the performance of challenged muscle groups [6, 7]. Contracting skeletal muscle can also modulate the function of metabolically relevant tissues with production and release of myokines [8]. The enhancement of muscle metabolic efficiency and crosstalk of muscle with other tissues constitute the fundamental ingredients by which physical exercise improves the health of whole organism [7]. One of the most relevant exercise-induced muscle remodeling responses, from the perspective of metabolic efficiency, is the increase in mitochondrial density and enzyme activity, termed mitochondrial biogenesis [6]. Mitochondrial biogenesis is a complex process that requires co-expression of genes from two distinct genomes (nuclear and mitochondrial) and is regulated by transcription factors and transcription co-activators [8].

Peroxisome proliferator receptor-γ co-activator-1α (PGC-1α) is an inducible transcription co-activator that interacts with many different transcription factors to activate distinct biological programs in a multitude of tissues. In skeletal muscle, PGC-1α is readily induced by endurance exercise and regulates the coordinated expression of mitochondrial proteins encoded in both nuclear and mitochondrial genomes. Induction of PGC-1α in skeletal muscle leads to activation of genetic programs characteristic of slow-twitch (type I, predominantly oxidative) muscle fibers and phenotypical changes such as increase in functional mitochondria, improvement in whole-body VO2_max_, shift of fuel usage from carbohydrate to fat during submaximal exercise, and improved endurance performance [9]. Moreover, PGC-1α mediates the exercise-dependent up-regulation of fibronectin type III domain-containing protein 5 (FNDC5) which is proteolytically cleaved to generate irisin—a myokine that enhances thermogenesis and promotes conversion of white adipose cell to brown adipose cell [10]. Therefore, exercise induction of PGC-1α seems to be an event that orchestrates adaptive responses of skeletal muscle to physical exercise although the results from loss-of-function studies suggest that PGC-1α is probably not mandatory for some of the training-induced adaptive responses [11–13].

Protein turnover—proteolysis coupled with de novo protein synthesis—is another cellular process involved with exercise-induced muscle remodeling. Damaged proteins and organelles need to be removed by proteasome and autophagy proteolysis and replaced by newly synthesized ones in exercised muscles during the recovery [14]. Autophagy also plays an essential role in maintaining the mass of skeletal muscle and provides skeletal muscle cells with an alternative energy source during energy stress caused by physical training [14, 15]. Interestingly, loss-of-function studies have shown that autophagy is required for exercise-dependent mitochondrial biogenesis and improvement of endurance capacity [14, 16].

Metabolic syndrome (MetS) and obesity have a long-known relationship with decreased muscle mass and strength. Morphological and functional alterations have been observed in skeletal muscle of obese or MetS subjects [17–19], and mice exposed to high-fat diet have decreased total muscle mass of hind limbs, muscle fiber diameter, muscle protein content, and grip strength [20]. Moreover, skeletal muscle myotubes from severely obese individuals are shown to have altered proteasome and autophagic proteolytic flux [21]. These findings suggest that MetS (or obesity) is associated with morphological and functional abnormalities of skeletal muscle which might be a consequence of MetS (or obesity) per se or caused, at least in part, by altered proteolytic pathways or other cellular processes due to dietary habits or physical inactivity.

Increased consumption of high-fructose corn syrup (HFCS) or sucrose via ingestion of ultra-processed food and sugar-sweetened beverages (SSB) has been linked to the obesity and diabetes epidemics in the USA [22]. Fructose is a major monosaccharide component of both HFCS and sucrose and has been considered as responsible for the metabolic effects of these sweeteners [22, 23]. The liver is the major site of fructose metabolism which breaks fructose down into metabolic intermediates that enter promptly the triose pool in a process that bypasses the rate-limiting phosphofructokinase step. The expansion of triose phosphate pool is responsible for metabolic adaptations to acute fructose load while the responses to long-term load will depend on enzymatic adaptation [23]. In the liver of fed animals, the increase in the flux through the glycolytic pathway leads to lactate production, activation of pyruvate dehyderogenase, and enhancement of oxidative pathway with carbon dioxide and ketone body production [23]. This metabolic milieu also favors esterification of non-esterified fatty acids (NEFAs) augmenting the liver production and secretion of very low density lipoprotein (VLDL) [23]. In starved animals, activation of gluconeogenesis enzymes leads to formation of glucose from fructose [23]. Long-term load of fructose causes the liver to form more glucose and glycogen from fructose and respond more intensely to the actions of fructose in promoting VLDL output. In adipose tissue, fructose impairs both glucose utilization and esterification of fatty acids. This raises NEFAs concentration and increases VLDL production. Increased concentration of triglyceride and NEFAs impairs glucose utilization in skeletal muscle [23]. The consequence is increased insulin resistance, hyperinsulinemia, and formation of a vicious cycle in which insulin resistance will stimulate the already increased VLDL production by the liver. Thus, chronic fructose feeding will produce metabolic derangement similar to those found in the MetS.

Ingestion of fructose or maltodextrin has been shown to suppress the exercise-induced glucose transporter type 4 (GLUT4) adaptive response in rat skeletal muscle [24]. Motivated by this work, we conducted the present study to investigate if ingestion of fructose can impair the expression of genes involved in post-exercise muscle remodeling which is our primary aim in this study. The secondary aim of this study is to assess the effects of fructose ingestion and physical training on expression of selected genes involved in protein degradation in skeletal muscle.

## Methods

### Animals and experimental protocol

Eight-week-old male Wistar rats were provided by the University of São Paulo School of Medicine’s Animal Facility which keeps the animals in cages with four to five animals and feeds them with standard chow from weaning to the moment they started the protocol. The animals were randomly allocated into the following groups: sedentary control (C, *n* = 6), exercise-only (E, *n* = 7), sedentary fructose (F, *n* = 8), and fructose + exercise (FE, *n* = 8) and treated accordingly for 8 weeks. The pre-treatment weight of the rats ranged from 194.64 to 342.0 g, and there was no inter-group difference (*F*(3, 29) = 2.23, *p* = 0.110). The animals were kept in cages with four to five animals under a 12-h light/dark cycle and were given ad libitum access to food and water. Standard chow (2990 kcal/kg) was given as a solid diet. The rats assigned to fructose treatment (F and FE groups) were given a 15% fructose solution as drinking solution. The fructose treatment began on the same day as the exercise training (see the next section). The quantity and volume of unconsumed food and fluid for each cage were verified each morning. The daily consumption of food and fluid was calculated as a difference between what was provided on previous day and what was left unconsumed. Due to limited quantity of tissue and blood samples, biochemical and molecular analyses were not performed in all the animals.

This study was approved by the Ethics Committee of University of São Paulo School of Medicine under the number 073/13, and all animal experiments were performed according to the procedures approved at our institution.

### Treadmill exercise protocol

The rats in exercise training groups were initially acclimatized to the treadmill (KT 400, Imbramed, RS, Brazil) for 3 days (10 min/day, 0.3 km/h). Afterwards, a maximal exercise capacity test was performed with an initial velocity of 0.3 km/h for 5 min followed by an increase of 0.1 km/h every 1.5 min until animal exhaustion which was defined as the moment when an animal sat at the lower end of the treadmill and was unresponsive to 10 gentle taps to continue running. Total test time, velocity, and distance were recorded for each rat. The rats were trained at moderate intensity (60% of maximal velocity achieved in exercise capacity test) for 60 min/day, 5 days a week for 8 weeks. After 8 weeks, the maximal exercise capacity test was repeated. One of us (NGG) oversaw personally all treadmill trainings and, whenever necessary, provided with stimulation to any animal that was running slower than the speed established by the treadmill. No electrical shock was applied to the animals throughout the training period.

### Tissue collection and biochemical analysis

The rats were euthanized 1 day after the last training session. After an overnight fast, the animals were anesthetized with intraperitoneal injection of 75 mg/kg ketamine and 10 mg/kg xilazine. Blood was collected by cardiac puncture. Following blood collection, the rats were euthanized by decapitation, and the quadriceps femoris was dissected and preserved in RNAlater (Ambion) while blood samples were centrifuged at 5000 rpm at 4 °C and the resulting serum samples transferred to a fresh microcentrifuge tube. Both muscle and serum specimens were stored at −80 °C until use.

Serum insulin levels were measured with an ELISA kit (Millipore) as per the manufacturer’s instructions. Serum triglyceride and glucose levels were measured by enzymatic colorimetric assay in the Cobas c111 analyzer (Roche Diagnostics).

We used the HOMA2 model [25, 26] to evaluate insulin resistance (HOMA2-IR), pancreatic beta cell reserve (HOMA2-%B), and insulin sensitivity (HOMA2-%S). The indexes were calculated with the Oxford HOMA calculator [27].

### RNA extraction and gene expression analysis

Total RNA from quadriceps muscle was isolated with TRI Reagent (Sigma-Aldrich) as per the manufacturer’s instructions. Genomic DNA was removed by treating the RNA samples with DNase I for 20–30 min at 37 °C. RNA was reversely transcribed into complementary DNA with a commercial kit (High Capacity cDNA Reverse Transcription Kit, ABI) as per the manufacturer’s instructions. Gene expression analysis was performed using quantitative real-time polymerase chain reaction in assay buffer which contains EvaGreen fluorescent dye (5× HOT FIREPol® EvaGreen® qPCR Mix Plus (ROX), Solis BioDyne, Tartu, Estonia) using the primers listed in Table 1. Relative gene expression was calculated using procedures reported previously [28], and cyclophilin A (CypA) was adopted as internal normalization control. A sample collected from an untreated control was used as a calibrator in all real-time PCR quantification experiments.

**Table 1:** Primer pairs used in real-time PCR

Gene	Forward	Reverse
PGC-1α1	GGACATGTGCAGCCAAGACTCT	CACTTCAATCCACCCAGAAAGCT
FNDC5	ATGAAGGAGATGGGGAGGAA	GCGGCAGAAGAGAGCTATGACA
CAMK IV	AGGAGACCTCCAGTATGGTGC	CTCCTCAGTCATGGGGTCCAT
NR4A3	TCAGCCTTTTTGGAGCTGTT	TGAAGTCGATGCAGGACAAG
ERRα	GCAGGGCAGTGGGAAGCTA	CCTCTTGAAGAAGGCTTTGCA
PPARδ	CTCCTGCTCACTGACAGATG	TCTCCTCCTGTGGCTGTTC
FoxO3A	GCAAGCCGTGTACCGTGGA	CGGGAGCGCGATGTTATCT
GLUT4	GCAGCGAGTGACTGGAACA	CCAGCCACGTTGCATTGTAG
Atg6/beclin1	TGAATGAGGGCGACAGTGAACA	GCATCTGGTTCTCTACACTCTTG
Atg7	GCTCCTCACTTTTTGCCAACA	GGAGCCACCACATCATTGC
Atg9	CAGTTTGACACTGAATACCAGCG	AATGTGGTGCCAAGGTGATTT
LC3	CGTCCTGGACAAGACCAAGT	AGTGCTGTCCCGAATGTCTC
Lamp-2	TGGCTCAGCTTTCCTTGTTTC	CATATAAGAACTTCCCAGAGGAGCAT
Atg12	CACCACTGCACCTGCCTCATTTTTAACTC	ATGGCACACATGGCTGAGGACTACTCTG
Ctsl1	CTATCGCCACCAGAAGCACA	AACCACACTGGCCCTGATTC
Murf-1/TRIM63	ACCTGCTGGTGGAGAACATC	CTTCGTGTTCCTTGCACATC
MAFBx/Atrogin-1	TGGGTGTATCGAATGGAGAC	TCAGCCTCTGCATGATGTTC
Bnip3	TTCCACTAGTACCTTTTGATGA	GAACACCGCATTTACAAAACAA
CypA	TATCTGCACTGCCAAGACTGAGTG	CTTCTTGCTGGTCTTGCCATTCC

### Statistical analysis

All data are presented as mean ± SEM. Normality of samples was assessed with Shapiro-Wilk test. Homoscedasticity (homogeneity of variances) was assessed with Fligner-Killeen test due to robustness of this test [29]. Since there was no violation of normality or homogeneity of variances, no transformation of original data was necessary. Differences among groups of weight, metabolic profile, and exercise capacity were assessed by analysis of variance (ANOVA). The status of fructose ingestion and exercise training were used as factors and factorial ANOVA was used to assess the effect of each treatment on gene expression. This study has a power of 0.34 when an effect size of 0.4 (a large conventional effect size according to Cohen [30]) is used in the calculation. All statistical analyses were performed in R version 3.3.1. Study power was calculated using R packages pwr and pwr2 [31, 32]. A value of *p* < 0.05 was considered statistically significant.

## Results

### Effect of fructose and exercise on food, water, and calorie intake

Food and water intake were measured daily. The animals were kept in cages with four or five rats; therefore, it was not possible to perform statistical analysis of food, water, and calorie intake, only the means were compared. The animals assigned to groups F and FE ingested less food than groups C and E. On the other hand, F and FE consumed more water than C and E, resulting in higher calorie intake in the former (data not shown). These data agree with previous study [33].

### Effect of fructose and exercise in body weight

The animals were weighted before the diet/exercise protocols started (week 0) and again after the end of the diet/exercise protocols (week 8). There was no difference between groups in pre-treatment weight (*F*(3, 29) = 2.23, *p* = 0.110). At the end of the 8th week, the rats in E group presented with the lowest while the rats in FE group with the highest body weight (Table 2). A main effect of fructose ingestion on body weight (*F*(1, 30) = 6.885, *p* = 0.01354) as well as an interaction between fructose and exercise (*F*(1, 30) = 7.791, *p* = 0.00905) were detected.

**Table 2:** Body weight and metabolic profile after week 8 of fructose ingestion or treadmill training

	Body weight (g)	Glucose (mg/dL)	Triglyceride (mg/dL)	Insulin (mUI/mL)	HOMA2-IR	HOMA2-%B	HOMA2-%S
C (6)	479.77 ± 8.87	168.43 ± 8.06	46.01 ± 6.42	17.13 ± 0.44	2.51 ± 0.06	53.19 ± 4.02	40.17 ± 1.07
E (7)	429.25 ± 8.11	160.29 ± 5.93	41.05 ± 1.49	17.31 ± 0.49	2.54 ± 0.09	52.75 ± 2.80	39.8 ± 1.47
F (8)	475.93 ± 12.93	191.14 ± 19.49	52.3 ± 6.45	18.13 ± 0.77	2.82 ± 0.17	53.28 ± 8.77	36.68 ± 2.33
FE (8)	493.82 ± 7.29	166.86 ± 5.87	62.76 ± 5.40	17.36 ± 0.92	2.54 ± 0.13	53.20 ± 2.32	40.33 ± 2.06

Results are presented as mean ± SEM. The numbers in parenthesis represent the number of animals included in the experiment. A main effect of fructose (*p* = 0.01354) and an interaction between fructose and exercise (*p* = 0.00905) on animals’ weight were observed. There is also an effect of fructose on serum triglycerides (*p* = 0.0418). See the text for more details

### Effect of fructose in metabolic profile of the animals

To assess the metabolic profile of the animals, after the end of the diet/exercise protocols, serum glucose, insulin, and triglyceride levels were measured. HOMA2 model was used to evaluate insulin resistance (HOMA2-IR), pancreatic beta cell reserve (HOMA2-%B), and insulin sensitivity (HOMA2-%S). No significant effect of fructose ingestion or exercise training was detected for serum insulin (respectively, *F*(1, 25) = 0.397, *p* = 0.534 and *F*(1, 25) = 0.121, *p* = 0.731), glucose levels (respectively, *F*(1, 25) = 1.226 *p* = 0.279 and *F*(1, 25) = 1.401, *p* = 0.248), HOMA2-IR (respectively, *F*(1, 25) = 1.042, *p* = 0.317 and *F*(1, 25) = 0.627, *p* = 0.436), HOMA2-%S (respectively, *F*(1, 25) = 0.488, *p* = 0.491 and *F*(1, 25) = 0.560, *p* = 0.461), or HOMA2-%B (respectively, *F*(1, 25) = 0.002, *p* = 0.963 and *F*(1, 25) = 0.002, *p* = 0.966). In contrast, there is a main effect of fructose ingestion on serum triglyceride levels (*F*(1, 25) = 4.601, *p* = 0.0418), Table 2.

### Effect of exercise in the physical conditioning

**Fig. 1:**
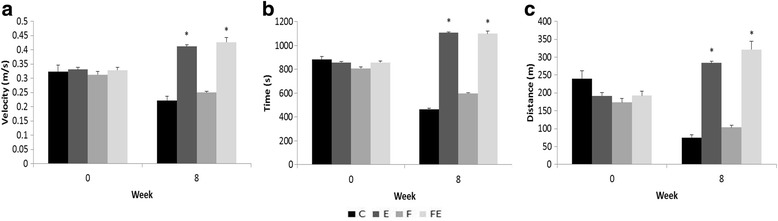
Maximal exercise capacity before (week 0) and after (week 8) 8 weeks of treadmill training or fructose ingestion. Groups: *C* control, *E* exercise, *F* fructose, *FE* fructose + exercise. Results are presented as mean ± SEM. **a** Velocity. **b** Duration of exercise. **c** Traveled distance. Exercised animals (E and FE) displayed higher exercise capacity than their non-exercised littermates (*p < 0.001) after 8 weeks in all parameters. E and FE rats also improved their exercise capacity at week 8 compared to week 0 (see text for more details)

To evaluate their physical conditioning, the animals underwent a maximal exercise capacity test before the diet/exercise protocols started and after the end of the protocols. In the initial maximal exercise capacity test, there was no statistical difference between the groups (*F*(3, 24) = 1.08, *p* = 0.379). After 8 weeks of treadmill training, groups E and FE were both able to run significantly faster than the non-trained groups C and F (*F*(3, 24) = 37.24, *p* < 0.001) and to reach higher speeds than they did during the initial test (E: *p* = 0.047; FE: *p* = 0.001). Interestingly, non-trained animals performed poorer in the final test relative to the initial test (C: *p* = 0.001; F: *p* = 0.035, Fig. 1a). The same trend is seen both in duration and distance. There were no between group differences regarding the duration of running (*F*(3, 24) = 0.59, *p* = 0.660) and traveled distance (*F*(3, 24) = 2.28, *p* = 0.090) at the initial assessment. After 8 weeks of training, both E and FE improved the duration (E: *p* < 0.001; FE: *p* = 0.001) and distance (E: *p* < 0.001; FE: *p* < 0.001). Both groups E and FE also ran for longer time (*F*(3, 24) = 58.66, *p* < 0.001) and a greater distance (*F*(3, 24) = 42.34, *p* < 0.001) than their littermates assigned to sedentary groups (C and F). The non-trained animals (groups C and F) also performed poorer relative to their own initial test in both duration (C: *p* = 0.003; F: *p* = 0.008, Fig. 1b) and traveled distance (C: *p* = 0.005; F: *p* = 0.009, Fig. 1c). This degradation of exercise capacity of C and F is probably a result of physical deconditioning that the untrained animals underwent after 8 weeks of sedentarism.

### Impact of fructose ingestion and exercise training on expression of PGC-1α and FNDC5

**Fig. 2:**
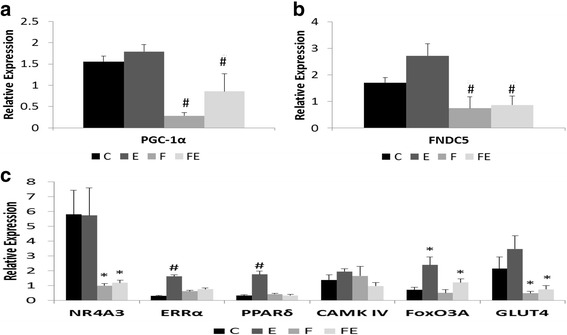
Impact of aerobic training or fructose ingestion on expression of genes involved in regulation of mitochondrial biogenesis in rat skeletal muscle. **a** PGC-1α1. **b** FNDC5. **c** Transcriptional regulators of skeletal muscle adaptive response to exercise training and GLUT4. Groups: *C* control, *E* exercise, *F* fructose, *FE* fructose + exercise. Relative expression was calculated using methods described by Livak and Schmittgen. Results are presented as mean ± SEM. A main effect of fructose on expression of PGC-1α1, FNDC5, NR4A3/Nor-1, and GLUT4 was detected. In addition to the main effect of fructose, there is also a main effect of exercise and an interaction fructose exercise on expression of Errα and Pparδ. In contrast, there is a main effect of exercise on expression of FoxO3A. (see the text for further details). * *p* < 0.040, # *p* < 0.001

Ingestion of fructose negatively affected expression of both PGC-1α and FNDC5 in rat skeletal muscle regardless of their exercise status. Fructose-ingesting sedentary rats exhibited a less intense expression of PGC-1α and FNDC5 than littermates that did not ingest fructose (Fig. 2a, b). Furthermore, expression of these two genes after exercise is also decreased in the fructose-fed animals when compared to the exercised animals that did not ingest fructose (Fig. 2a, b). Indeed, fructose was the only treatment that affected the expression of both PGC-1α and FNDC5 (*F*(1, 9) = 11.720, *p* = 0.00759 and *F*(1, 9) = 11.310, *p* = 0.00835, respectively, PGC-1α and FNDC5).

To gain further insight on the effects of fructose ingestion on molecular mediators of beneficial effects of exercise training, we studied the expression of transcription factors nuclear receptor subfamily 4 group A member 3 (NR4A3/Nor-1), estrogen-related receptor alpha (Errα), and peroxisome proliferator activated receptor δ (Pparδ) which are induced in skeletal muscle by endurance exercise. Although average expression of NR4A3/Nor-1 was higher in rats undergoing physical training, no statistically significant effect of treadmill running was observed among rats that did not ingest fructose. In contrast, fructose-treated (F and FE) rats exhibited a 80% decrease in expression of NR4A3/Nor-1 when compared to the littermates that did not ingest fructose (C and E groups, *p* = 0.027, Fig. 2c). In fact, an effect of fructose on expression of NR4A3/Nor-1 was noted (*F*(1, 8) = 7.651, *p* = 0.0244). Expression of Errα and Pparδ exhibited a very similar pattern. Both were strongly induced by treadmill running in skeletal muscle (79 and 66%, respectively, Errα and Pparδ, Fig. 2c). There are main effects of fructose ingestion, exercise training, and interaction between fructose and exercise on expression of both Errα (respectively, *F*(1, 11) = 17.61, *p* = 0.001494, *F*(1, 11) = 47.38, *p* = 2.64 × 10^−05^, and *F*(1, 11) = 29.61, *p* = 0.000203) and Pparδ (respectively, *F*(1, 10) = 20.43, *p* = 0.00111, *F*(1, 10) = 14.54, *p* = 0.00341, and *F*(1, 10) = 19.26, *p* = 0.00136). We also assessed how fructose ingestion affects expression of calcium/calmodulin-dependent protein kinase type IV (CAMK IV) in skeletal muscle as this kinase is reported to transduce muscle contraction into a regulatory signal for the expression of PGC-1α [34]. No effect of fructose ingestion or treadmill training on expression of CAMK IV was observed (*F*(1, 11) = 0.712, *p* = 0.417 and *F*(1, 11) = 0.001, *p* = 0.982; Fig. 2c). We also assayed the expression of GLUT4 which is induced by exercise training and is responsible for the enhanced muscle glucose uptake caused by chronic exercise. Fructose ingestion attenuated expression of GLUT4 by 78% in skeletal muscle of either sedentary or exercised rats (*p* = 0.0156, Fig. 2c), and a significant main effect of fructose ingestion on GLUT4 expression was detected (*F*(1, 12) = 5.848, *p* = 0.0324). Therefore, ingestion of fructose globally attenuates expression of key genes involved in metabolic adaptation of skeletal muscle to physical exercise.

Expression of forkhead box O3 (FoxO3A)—a transcriptional factor reported to interact with PGC-1α1 to regulate expression of oxidative stress genes [35]—was also assessed in the skeletal muscle. While fructose showed no effect (*F*(1, 16) = 2.055, *p* = 0.1710; Fig. 2c) a main effect of aerobic training on the expression of FoxO3A was detected (*F*(1, 16) = 5.711, *p* = 0.0295).

### Expression of genes involved in protein degradation

The results on the expression of PGC-1α and FNDC5 and their transcriptional regulators led us to seek whether fructose ingestion might affect other molecular pathways that also mediate adaptive metabolic response of skeletal muscle to physical exercise. Should this be the case attenuation of exercise-induced remodeling of skeletal muscle might be, in addition to excessive caloric accumulation, a relevant mechanism underlying metabolic derangement associated with fructose ingestion. Autophagy and ubiquitin-proteasome pathways are major protein degradation pathways in the skeletal muscle. In addition to regulating the net amount and the quality of muscle protein, autophagy (basal and acute, exercise-induced) has been shown to play a critical role in exercise-induced muscle remodeling and improvement of insulin sensitivity [16, 36].

**Fig. 3:**
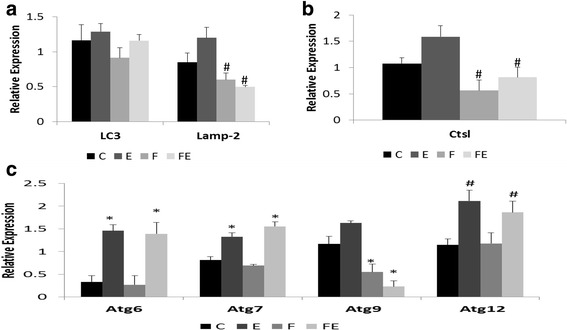
Impact of aerobic training or fructose ingestion on expression of autophagy genes. **a** Marker of autophagosome accumulation LC3 and lysosomal-associated membrane glycoprotein Lamp-2. **b** Lysosomal cathepsin L Ctsl. **c** Atg genes. Groups: *C* control, *E* exercise, *F* fructose, *FE* fructose + exercise. Results are presented as mean ± SEM. A statistically significant main effect of exercise on expression of Atg6, Atg7, and Atg12 was observed. There is also a main effect of fructose ingestion on expression of Atg9, Lamp-2, and Ctsl as well as an interaction between fructose and exercise on expression of Atg9. See the text for further details. * *p* < 0.001, # *p* < 0.030

**Fig. 4:**
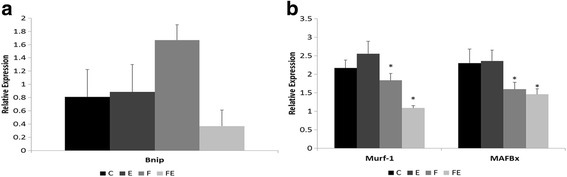
Impact of fructose ingestion and exercise on expression of **a** mitophagy marker Bnip3 and **b** E3 ubiquitin ligases Murf-1 and MAFBx. Groups: *C* control, *E* exercise, *F* fructose, FE fructose + exercise. Results are presented as mean ± SEM. There is a main effect of fructose ingestion on expression of Murf-1 and MAFBx (see the text for more details)

We observed a statistically significant main effect of exercise training on expression of autophagy-related protein 6 (Atg6/beclin 1) (*F*(1, 11) = 23.856, *p* = 0.000484), autophagy-related protein 7 (Atg7) (*F*(1, 13) = 27.609, *p* = 0.000156), and autophagy-related protein 12 (Atg12) (*F*(1, 15) = 8.157, *p* = 0.012), and they all showed significant induction in skeletal muscle after treadmill running (Fig. 3c). No significant effect for fructose ingestion or interaction between exercise and fructose ingestion was observed on expression of these genes. In contrast, autophagy-related protein 9 (Atg9) expression in both sedentary and exercised rats was impaired by fructose ingestion (Fig. 3c), and there was a significant main effect of fructose (*F*(1, 14) = 28.972, *p* = 9.66 × 10^−05^) and interaction between exercise and fructose (*F*(1, 14) = 4.653, *p* = 0.0489) on expression of Atg9. Expression of microtubule-associated protein 1 light chain 3 isoform B (LC3B)—a marker of autophagosome accumulation was not affected by physical training or fructose (respectively, *F*(1, 20) = 0.951, *p* = 0.341 and *F*(1, 20) = 0.811, *p* = 0.378; Fig. 3a). However, ingestion of fructose impaired expression of lysosome-associated membrane protein 2 (Lamp-2, *F*(1, 17) = 7.750, *p* = 0.0127; Fig. 3a). Fructose ingestion also attenuated expression of lysosomal cathepsin L (Ctsl) in skeletal muscle (*F*(1, 14) = 6.768, *p* = 0.0209, Fig. 3b). No statistically significant main effect for exercise or fructose on expression of BCL2/adenovirus E1B interacting protein B (Bnip3)—a marker of mitochondrial autophagy—was detected except for a trend for interaction between exercise and fructose (*F*(1, 11) = 3.401, *p* = 0.0923, Fig. 4a).

Effects of exercise and fructose ingestion on ubiquitin-proteasome pathway were also evaluated by studying the expression of E3 ubiquitin ligases muscle RING-finger protein-1 (Murf-1) and muscle atrophy F-box (MAFBx, also known as atrogin-1) (Fig. 4b). There was a main effect of fructose on expression of both Murf-1 (*F*(1, 19) = 12.181, *p* = 0.00245) and MAFBx (*F*(1, 17) = 4.897, *p* = 0.0409) and a marginally significant interaction between exercise and fructose on expression of Murf-1 (*F*(1, 19) = 4.000, *p* = 0.05999).

## Discussion

The main finding of this study is that fructose ingestion impairs the expression of genes involved in transcriptional regulation of both oxidative metabolism and mitochondrial biogenesis and of genes of proteolytical pathways in the skeletal muscle. This negative effect of fructose ingestion was seen in both sedentary and exercised animals for most of these genes, but a few of these genes showed blunted expression only in treadmill-trained animals. Our results not only confirm the finding of a previous work which reported that fructose consumption impairs adaptive response of GLUT4 [24] but also suggest that ingestion of fructose might impair other responses of skeletal muscle to exercise.

Our results are similar to a recent study with human volunteers in which failure to upregulate mitochondrial fuel oxidation genes was shown as the mechanism behind the inability of human subjects to improve their insulin sensitivity upon aerobic training [37]. Like our study, the skeletal muscle of those who were unable to respond to aerobic training displayed deficient exercise-induced expression of PGC-1α, ERRα, and of 5′-AMP-activated protein kinase catalytic subunit alpha-2 (AMPKα2) [37]. This study and ours highlighted the importance of oxidative muscle fibers in the genesis of insulin resistance and related metabolic diseases. Decreased oxidative phosphorylation in skeletal muscle has been reported as the earliest defect leading to insulin resistance and glucose intolerance in elderly subjects and non-diabetic offspring of type 2 diabetes patients [38, 39]. In fact, the latter group also displayed a reduced ratio of inorganic phosphate to phosphocreatine in soleus muscle which is compatible with a diminished content of type I (oxidative) fibers relative to type II fibers [39]. Content of type I fibers has also been shown to correlate inversely with fat body mass and positively with the response to weight loss intervention [40]. Therefore, our results open the possibility that a dietary factor might lead to disorders associated to insulin resistance via reduction of number or function of mitochondria in skeletal muscle.

Interestingly, in our study, fructose feeding also prevented exercise induction of selected autophagy genes and muscle-specific E3 ubiquitin-protein ligases Murf-1 and MAFBx. A functioning autophagy pathway seems to be required for muscle mass maintenance, muscle regeneration, and exercise-induced muscle remodeling [14, 16, 36] while both expression of E3 ligases and proteasome activity in skeletal muscle have been reported to increase with either acute or chronic endurance exercise [14]. Such activation of proteasomal proteolysis might be an adaptive response as it allows for removal of damaged proteins and facilitates myofilament restructuring [14]. Therefore, fructose ingestion seems to affect multiple cellular functions that are related to skeletal muscle remodeling and metabolic adaptation to endurance training.

Intriguingly, in the present study, the fructose-loaded rats that underwent treadmill training (FE group) improved their exercise capacity to a similar extent as did their exercise-only counterparts (E group). Such finding is not what one might predict considering the altered gene expression exhibited by FE rats and evidences from overexpression experiments regarding the effects of PGC-1α on mitochondrial biology and energy metabolism. Possible explanations include the existence of other transcriptional co-activators that might provide redundancy for PGC-1α signaling or that PGC-1α might not be mandatory for some of training-induced adaptations. In fact, PGC-1α is a prototypical member of a family of transcriptional coactivators that regulates mitochondrial biogenesis and energy production, and there seems to be a redundancy between members of this family [41, 42]. Also, loss-of-function studies have shown that PGC-1α might not be mandatory for some of training-induced responses in skeletal muscle [11, 13, 43]. It is noteworthy that in the study by Bohm et al. [37], no group of volunteers showed significant training-related improvement of VO2_max_ regardless of their ability (or inability) to improve insulin sensitivity with aerobic exercise or to induce expression of PGC-1α, ERRα, and AMPKα2.

One might speculate the mechanism underlying the defective induction of genes related to skeletal muscle response to aerobic training in fructose-fed animals. These observed effects of fructose ingestion are probably mediated by transcriptional mechanism as the affected genes encompass multiple cellular processes. Cyclooxygenase 2-mediated inflammation have been reported to be the underlying mechanism of fructose-induced insulin resistance in rats [44, 45]. A persistent inflammation caused by fructose ingestion might lead to defective activation of PGC-1α and other transcriptional regulators of skeletal muscle adaptation via a TGFβ-dependent mechanism like the one underlying the defective activation of PGC-1α and AMPKα2 in individuals who failed to improve insulin sensitivity upon aerobic training [37]. This hypothesis, however, contradicts the existing evidence of anti-inflammatory properties of chronic aerobic exercise in rodent models of diabetes and tobacco smoking [46–48]. Alternatively, fructose might impair exercise-induced skeletal muscle remodeling by interfering with post-exercise glycogen accumulation in skeletal muscle. Exercise-induced activation of Pparδ—a known activator of PGC-1α transcription [49]—varies inversely with the glycogen content of muscle fiber [50]. Also, exercise-trained rats that ingest fructose exhibit higher content of both liver and muscle glycogen content than their exercise-trained, control diet-fed littermates [51]. Therefore, ingestion of fructose might impair the activation of Pparδ and its downstream transcription targets including PGC-1α by enhancing the accumulation of glycogen in skeletal muscle. Whether Pparδ functions as an upstream transcriptional regulator of proteolytic pathways remains to be determined. Fructose ingestion might also affect expression PGC-1α and training-induced adaptive genes responses by promoting the accumulation of lactate or lipids. In the liver, where most of absorbed fructose is metabolized, fructose is first phosphorylated by fructokinase to form fructose-1-phosphate then broken down to glyceraldehyde and dihydroxyacetone phosphate by aldolase B [23]. The glyceraldehyde thus generated is phosphorylated to glyceraldehyde-3-phosphate by triokinase after which it can follow any triose phosphate metabolic pathway including conversion to lactate [23, 52]. Conversion to lactate is a means to release fructose-derived carbon from liver for extrahepatic utilization, and about a quarter of ingested fructose is converted to lactate [53]. Thus, lactate might be a fructose-derived metabolic intermediate that causes the muscle to impair exercise-induced gene response. The caveat for this hypothesis is the fact that exposure to lactate has been reported to promote expression of PGC-1α and genes involved in mitochondrial biogenesis in both cultured L6 cells [54] and C57BL/6J mice [55]. Finally, excessive exposure of skeletal muscle to lipids results in muscle insulin-resistance and accumulation within muscle fiber of fatty acid metabolites [56]. Since, by both augmenting lipid synthesis and decreasing lipid clearance, fructose loading increases plasma triglyceride and NEFAs [23, 53], metabolic overload of skeletal muscle mitochondria might impair the training-induced gene expression in skeletal muscle.

This study presents a number of limitations that should be mentioned. Firstly, we did not include isocaloric controls of other sugar preparations. For this reason, we could not test whether the observed effects on gene expression is specific to fructose ingestion or is a general phenomenon associated to excessive carbohydrate (or caloric) consumption. Second, animal’s insulin sensitivity status was assessed only after the treatment/training period, and this hinders inferences that can be made regarding the effect of training or fructose on insulin sensitivity. Third, we used maximal exercise capacity on treadmill running to evaluate the effect of training instead of VO2_max_. Since exercise capacity is determined by a combination of factors which include VO2_max_ [57], we might not have captured adequately the impact of altered gene response on the physiology of skeletal muscle. Fourth, we did not allow the animals in this study to perform voluntary physical activity. For this reason, it is possible that our test for exercise capacity was comparing physical conditioning with physical deconditioning. The latter two deficiencies of our study might also be the reason why no apparent difference in exercise capacity between E and FE animals was detected. Finally, in view of the limited power of this study, we might have failed to detect an effect of fructose or exercise. To assess how the design of this study would affect our ability to detect an effect of treatment factors on gene expression, we calculated the power using as parameters the effect sizes obtained from our PGC-1α and FNDC5 expression data (Table 3). Post hoc power analysis showed that the power of this study to detect an effect of fructose on PGC-1α or FNDC5 is 0.8 but only 0.05 for effect of exercise on either gene. Importantly, we were able to detect interaction between fructose and exercise on expression of a few genes despite of small effect size attributable to exercise (Table 3). In our opinion, the limitations mentioned here do not invalidate the main conclusion of our study regarding the possibility of excessive ingestion of a macronutrient impairing beneficial adaptive responses in skeletal muscle.

**Table 3:** Effect size of treatments (fructose or exercise) on expression of genes involved in skeletal muscle metabolic adaptation

Gene	Effect size of fructose	Effect size of exercise
Pgc1alpha/irisin pathway		
PGC-1α	0.553419169	0.003713567
FNDC5	0.548825642	0.000276319
Genes involved in muscle metabolic adaptation		
NR4A3	0.488632964	7.47831 × 10^−05^
Errα	0.166772854	0.448667531
PPARδ	0.317994819	0.226420844
CAMK IV	0.052895965	3.55006 × 10^−05^
FoxO3A	0.083590432	0.232332765
GLUT4	0.307669308	0.044401256
Genes involved in regulation of autophagy and proteasome pathways		
Atg6	0.00014622	0.684310572
Atg7	0.025548069	0.634666028
Atg9	0.606486173	0.003040394
Atg12	0.013919095	0.343627664
LC3	0.037156357	0.043599656
Lamp2	0.283530513	0.031508723
Ctsl1	0.290616142	0.096971505
Murf1	0.341522128	0.013641133
MAFBx	0.222860389	0.000315577
Bnip	0.029478458	0.132199546

$\eta 2\left({SS}_{Effect}{/}_{SS_{Total}}\right)$ was used as an estimate of effect size

## Conclusion

Our results suggest that fructose might impair exercise induction of genes involved in regulation of metabolic adaptation of the skeletal muscle. This finding indicates the need for a more detailed examination of the role of diet-exercise interaction in the pathophysiology of cardiometabolic diseases. Further studies are needed to elucidate the mechanisms underlying the impairment of skeletal muscle metabolic adaptation induced by fructose consumption.

## Abbreviations

AMPKα2: 5′-AMP-activated protein kinase catalytic subunit alpha-2
Atg12: Autophagy-related protein 12
Atg6/beclin 1: Autophagy-related protein 6
Atg7: Autophagy-related protein 7
Atg9: Autophagy-related protein 9
Bnip3: BCL2/adenovirus E1B interacting protein B
CAMK IV: Calcium/calmodulin dependent protein kinase type IV
Ctsl: Lysosomal cathepsin L
CypA: Cyclophilin A
Errα: Estrogen-related receptor alpha
FNDC5: Fibronectin type III domain-containing protein 5
FoxO3A: Forkhead box O3A
GLUT4: Glucose transporter type 4
Lamp-2: Lysosome-associated membrane protein 2
LC3B: Microtubule-associated protein 1 Light Chain 3 Isoform B
MAFBx/atrogin-1: Muscle atrophy F-box
Murf-1: Muscle RING-finger protein-1
NR4A3/Nor-1: nuclear receptor subfamily 4 group A member 3
PGC-1α: Peroxisome proliferator receptor-γ co-activator-1α
Pparδ: Peroxisome proliferator activated receptor δ
